# Comparison of the prognostic difference between ypTNM and equivalent pTNM stages in esophageal squamous cell carcinoma based on the 8th edition of AJCC classification

**DOI:** 10.7150/jca.34567

**Published:** 2020-01-20

**Authors:** Dongxian Jiang, Hao Wang, Qi Song, Haixing Wang, Qun Wang, Lijie Tan, Yingyong Hou

**Affiliations:** 1 Department of Pathology, Zhongshan Hospital, Fudan University, Shanghai 200032, P. R. China,; 2 Department of Thoracic Surgery, Zhongshan Hospital, Fudan University, Shanghai 200032, P. R. China,; 3 Department of Pathology, School of Basic Medical Sciences & Zhongshan Hospital, Fudan University, Shanghai 200032, P. R. China,; 4 Department of Pathology, Qingpu Branch of Zhongshan Hospital, Fudan University, Shanghai 201700, P. R. China

**Keywords:** ypTNM stages, equivalent pTNM stages, prognostic difference, esophageal squamous cell carcinoma (ESCC), 8th edition of AJCC classification

## Abstract

**Objective:** With the separate ypTNM stage groupings established in the 8th edition of AJCC staging system for esophageal squamous cell cancer (ESCC), we aimed to evaluate the prognostic difference between ypTNM stage and equivalent pTNM stage.

**Methods:** ESCC patients with surgery alone (cohort 1) and patients with neoadjuvant therapy plus surgery (cohort 2) were enrolled in the study.

**Results:** In p0, pIb, pIIa, pIIb, pIIIa, pIIIb and pIVa stages of cohort 1, the 5-year DFS and OS rates were 100/100%, 80.5/86.2%, 58.9/57.8%, 51.1/52.7%, 36.3/35.8%, 21.5/22.6% and 11.9/18.0%. In ypI, ypII, ypIII and ypIVa stages of cohort 2, the 5-year DFS and OS rates were 60.9/67.0%, 44.3/52.1%, 48.4/43.2% and 0. Patients in ypI stage had a tendency of poorer survival compared with those in pI stage (*P*=0.024 for DFS, *P*=0.067 for OS). There was no significant difference in terms of DFS (*P*=0.335) or OS (*P*=0.903) between ypII and pII. Patients in ypIII stage had a tendency of better survival compared with those in pIII stage (*P*=0.015 for DFS, *P*=0.059 for OS). Patients in ypIVa stage exhibited a significantly poorer OS compared with those in pIVa stage (*P*=0.038).

**Conclusions:** With down-staged tumor after neoadjuvant therapy, survival of ypI was closed but not reached to the prognosis of equivalent pI, prognosis of ypII was similar to equivalent pII, and survival of ypIII tended to be better compared with equivalent pIII. However, without down-staged ypIVa tumor, the prognosis was worse compared with equivalent pIVa, indicating those patients were primary resistant to prescribed neoadjuvant therapy.

## Introduction

Esophageal cancer (EC) is the ninth most commonly diagnosed cancer and the sixth most common cause of cancer death globally^1^. Within the so-called EC belt, China alone contributes more than half of the global EC cases^2^, ^3^. The Chinese National Central Cancer Registry (NCCR) called for data in 2015 from all population-based cancer registries and showed that EC, with estimated new cases of 477,900 and death of 375,000, ranks as the third most common cancer and the fourth leading cause of cancer death in China^4^. The overall 5-year survival rate of EC ranges from 15% to 25%^5^. The better outcomes are associated with early diagnosis. However, many Chinese patients are diagnosed in locally advanced stages with poorer outcomes^6^. Therefore, it remains a challenge to improve the survival of patients with locally advanced tumor in China.

In recent years, neoadjuvant therapy, including chemotherapy and/or radiotherapy, is commonly used as an adjunct to surgical resection in patients with locally advanced EC, leading to down-staged tumor, increased resection rate and improved survival^1^, ^7^, ^8^. With the widespread use of neoadjuvant therapy, it clearly creates an extra demand on prognostic evaluation. The 7th edition of American Joint Committee on Cancer (AJCC) staging system is widely used for prognosis stratification throughout the world in EC patients with surgery alone^9^-^11^. Some studies believe that the ypTNM classification describing the presence and anatomical extent of vital tumor cells is also an essential prognostic factor in EC patients with neoadjuvant therapy^12^, ^13^. However, many investigators have argued that this ypTNM stage largely loses its prognostic strength in EC patients with neoadjuvant therapy^14^, ^15^, partly because that the database which is used to establish the 7th edition of AJCC ypTNM staging system includes patients who undergo surgical resection alone and excludes patients who receive neoadjuvant therapy followed by surgery.

An 8th edition primer of the AJCC staging system for EC was published in January 2017^16^. New to the 8th edition is stage grouping of 7,773 patients treated with neoadjuvant therapy from 33 Worldwide Esophageal Cancer Collaboration (WECC) institutions, and the separate stage groupings are established for patients with neoadjuvant therapy^17^, ^18^. With the new TNM staging system, the intriguing question is whether there is prognostic difference between neoadjuvant categories (ypTNM) and equivalent pathologic categories (pTNM). At present, no related data comparisons have been reported using the new staging system.

In the present study, we presented data from 779 esophageal squamous cell carcinoma (ESCC) patients who underwent surgery (cohort 1) or received neoadjuvant therapy plus surgery (cohort 2) from a single cancer center. We aimed to evaluate the prognostic difference between ypTNM stage in cohort 2 and equivalent pTNM stage in cohort 1 based on the 8th edition of AJCC staging system.

## Materials and Methods

### Patient selection

A total of 779 patients with biopsy-proven primary ESCC who were treated with curative intent at Zhongshan Hospital, Fudan University were enrolled in this retrospective analysis. According to the different treatment modalities, these patients were divided into two groups (cohort 1 and 2). All patients included for analysis fit the following inclusion criteria: 1) pathologically confirmed ESCC; 2) received R0 resection; 3) without neoadjuvant therapy for cohort 1; with neoadjuvant therapy for cohort 2. The main exclusion criteria were past or current history of malignancy other than the ESCC, and lost in follow-up. In cohort 1, a total of 604 patients only underwent surgical resection without neoadjuvant therapy from January 2007 to November 2010. In cohort 2, a total of 175 patients with locally advanced EC received neoadjuvant therapy (neoadjuvant chemotherapy or radiochemotherapy) in combination with surgical resection from November 2009 to December 2015.

Patients were initially diagnosed through endoscopy, and tumor staging was confirmed with computed tomography (CT) scan of the thorax and abdomen. Endoscopic ultrasound (EUS) and positron emission tomography (PET) were not routinely used. Patients were followed up with a clinical examination every 3 months for the first year, every 6 months for the second year, and every 6-12 months thereafter. Patients who did not go to our hospital were contacted by telephone to obtain follow-up data.

This study was approved by the Institutional Review Board of Zhongshan Hospital, Fudan University in accordance with the Declaration of Helsinki. Informed consent was obtained from all patients before treatment.

### Clinicopathologic evaluation

All hematoxylin and eosin-stained glass slides were reviewed by two experienced pathologists. Following clinicopathologic features were collected, including tumor grade (G category), invasive depth (pT category or ypT category), the number of positive lymph nodes (pN category or ypN category), and the presence of vessel (lymphatic and venous) invasion and nerve invasion. Other demographic characteristics, including age, gender and tumor location, were also recorded.

### TNM staging

Tumors were staged into various pathologic stage groups (pTNM) as follows according to the 8th edition of the AJCC staging system for ESCC^19^: pStage 0 (pTis), pStage IA (pT1aN0M0G1) and pStage IB (pT1aN0M0G2-3, pT1bN0M0 and pT2N0M0G1), pStage IIA (pT2N0M0G2-3 cancers, pT3N0M0 cancers of the lower thoracic esophagus and pT3N0M0G1 cancers of the upper middle thoracic esophagus), pStage IIB (pT3N0M0G2-3 cancers of the upper middle thoracic esophagus and pT1N1M0 cancers), pStage IIIA (pT2N1M0 and pT1N2M0), pStage IIIB (pT2N2M0, pT3N1-2M0 and pT4aN0-1M0) and pStage IVA (pT4aN2M0, pT4bN0-2M0 and pTanyN3M0) (Table 1).

Post-neoadjuvant pathologic stage groups (ypTNM) included ypStage I (ypT0-2N0M0 cancers), ypStage II (ypT3N0M0), ypStage IIIA (ypT0-2N1M1), ypStage IIIB (ypT1-3N2M0, ypT3N1M0 and ypT4aN0M0 cancers) and ypStage IVA (ypT4aN1-2M0, ypT4bN0-2M0 and ypTanyN3M0)^17^ (Table 1).

### Statistical analyses

Disease-specific overall survival (OS) was calculated from the date of operation to the date of death or most recent follow-up. For tumor specific survival, an event was defined by death of ESCC. For disease free survival (DFS), the first occurrence of locoregional recurrence, distant metastasis or death by ESCC was defined as an event. OS and DFS were estimated using the Kaplan-Meier method. The log rank test was used to compare survival curves.

All statistical analyses were performed using the statistical program SPSS, version 21.0 (SPSS Inc., Chicago, IL, USA). *P* values of less than 0.05 were considered statistically significant.

## Results

### Patient demographics

Table 2 lists clinical characteristics of patients. In cohort 1, there were 488 males and 116 females with a median age of 61 years (range, 33-84 years). By anatomic site, 54.1% tumors were located in the middle esophagus, while 4.5% and 41.4% were located in the upper and lower esophagus, respectively. Among 600 invasive tumors, 360 (59.6%) cases were histologically graded as well to moderately differentiated, and 240 (39.7%) cases were poorly differentiated. Vessel and nerve invasion were identified in 131 (21.7%) and 200 (33.1%) tumors, respectively. Lymph node metastasis was observed in 38.9% (235/604) of patients, 136 patients (22.5%) had 1-2 positive lymph nodes, 78 patients (12.9%) had 3-6 positive lymph nodes, and 21 patients (3.5%) had more than 6 positive lymph nodes.

In cohort 2, there were 148 males and 27 females with a median age of 60 years (range, 41-73 years). By anatomic site, 46.9% tumors were located in the middle esophagus, while 16.6% and 36.6% in the upper and lower esophagus, respectively. Vessel and nerve invasion were identified in 57 (32.6%) and 56 (32.0%) tumors, respectively. Lymph node metastasis was observed in 47.4% (83/175) of patients, 51 patients (61.5%) had 1-2 positive lymph nodes, 23 patients (27.7%) had 3-6 positive lymph nodes, and nine patients (10.8%) had more than 6 positive lymph nodes.

### TNM staging

Table 2 lists the distribution of pTNM and ypTNM according to the 8th edition of AJCC staging system.

In cohort 1, the number of patients in pTis, pT1a, pT1b, pT2, pT3 and pT4a stages was 4 (0.7%), 20 (3.3%), 54 (8.9%), 140 (23.2%), 385 (63.7%) and 1 (0.2%), respectively. The number of patients in pN0, pN1, pN2 and pN3 stages was 369 (61.1%), 136 (22.5%), 78 (12.9%) and 21 (3.5%), respectively. The number of patients in p0, pIA, p1B, pIIA, pIIB, pIIIA, pIIIB and pIVa stages was 4 (0.7%), 1 (0.2%), 68 (11.3%), 175 (29.0%), 132 (21.9%), 23 (3.8%), 180 (29.8%) and 21 (3.5%), respectively.

In cohort 2, the number of patients in ypT0, ypT1, ypT2, ypT3 and ypT4a stages was 26 (14.9%), 30 (17.1%), 36 (20.6%), 80 (45.7%) and 3 (1.7%), respectively. The number of patients in ypN0, ypN1, ypN2 and ypN3 stages was 92 (52.6%), 51 (29.1%), 23 (13.1%) and 9 (5.1%), respectively. The number of patients in ypI, ypII, ypIIIA, ypIIIB and ypIVa stages was 62 (35.4%), 30 (17.1%), 23 (13.1%), 50 (28.6%) and 10 (5.7%), respectively.

### Survival analysis

**Figure 1:**
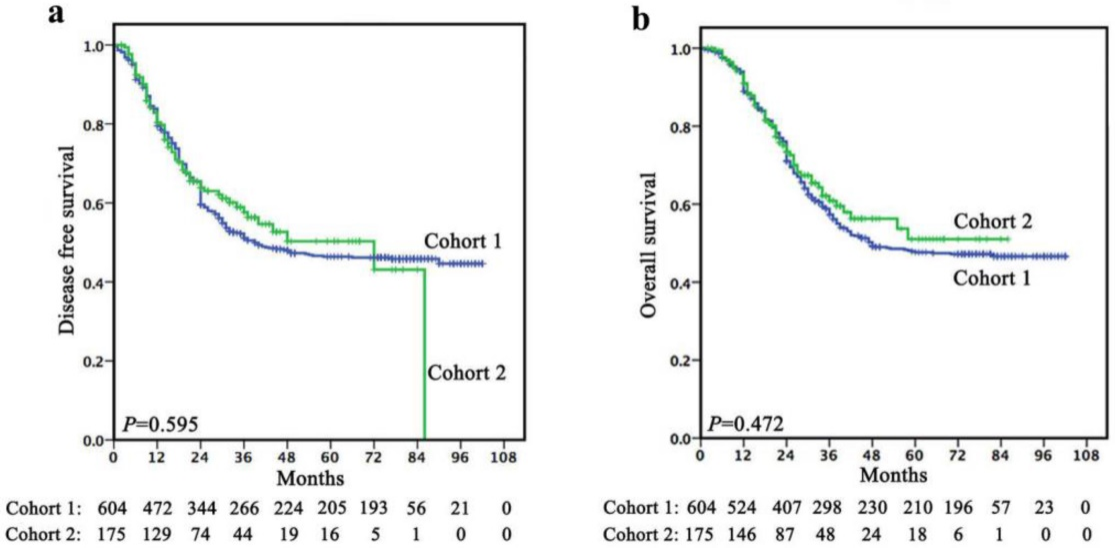
There was no significant difference in terms of survival between cohort 1 and cohort 2 (a, DFS; b, OS).

Follow-up was continued up to July 2017 or until death whichever occurred earlier. In cohort 1, the mean follow-up was 45.5 months (range, 1-102 months). The 1-, 3- and 5-year DFS rates were 79.5%, 51.1% and 46.4%, respectively, with a median survival time of 39.0 months (95% CI, 17.357-60.643). The 1-, 3- and 5-year OS rates were 88.9%, 57.3% and 47.6%, respectively, with a median survival time of 48.0 months. In cohort 2, the mean follow-up was 29.4 months (range, 2-86 months). The 1-, 3- and 5-year DFS rates were 80.4%, 57.6% and 50.3%, respectively, with a median survival time of 21.0 months. The 1-, 3- and 5-year OS rates were 91.0%, 60.9% and 51.0%, respectively, with a median survival time of 24.0 months. There was no significant difference in terms of survival between cohort 1 and cohort 2 (Figure 1).

### Survival comparisons within “the same stage”

In p0 stage (cohort 1), the 1-, 3- and 5-year DFS and OS rates were all 100%. In pI stage (cohort 1), the 1-, 3- and 5-year DFS and OS rates were 95.7/100%, 82.4/91.2% and 80.8/86.4%, respectively. In pII stage (cohort 1), the 1-, 3- and 5-year DFS and OS rates were 86.5/92.7%, 59.7/66.8% and 55.4/55.5%, respectively. In pIII stage (cohort 1), the 1-, 3- and 5-year DFS and OS rates were 64.8/80.1%, 29.1/33.1% and 23.1/23.9%, respectively. In pIVa stage (cohort 1), the 1-, 3- and 5-year DFS and OS rates were 61.9/80.2%, 23.8/30.1% and 11.9/18.0%, respectively (Table 3).

In ypI stage (cohort 2), the 1-, 3- and 5-year DFS and OS rates were 91.7/96.7%, 76.4/80.3% and 60.9/ 67.0%, respectively. In ypII stage (cohort 2), the 1-, 3- and 5-year DFS and OS rates were 89.7/100%, 51.7/ 66.1% and 44.3/52.1%, respectively. In ypIII stage (cohort 2), the 1-, 3- and 5-year DFS and OS rates were 73.2/85.7%, 48.4/46.8% and 48.4/43.2%, respectively. In ypIVa stage (cohort 2), the 1-year DFS and OS rates were 16.9% and 57.1%, respectively (Table 3).

**Figure 2:**
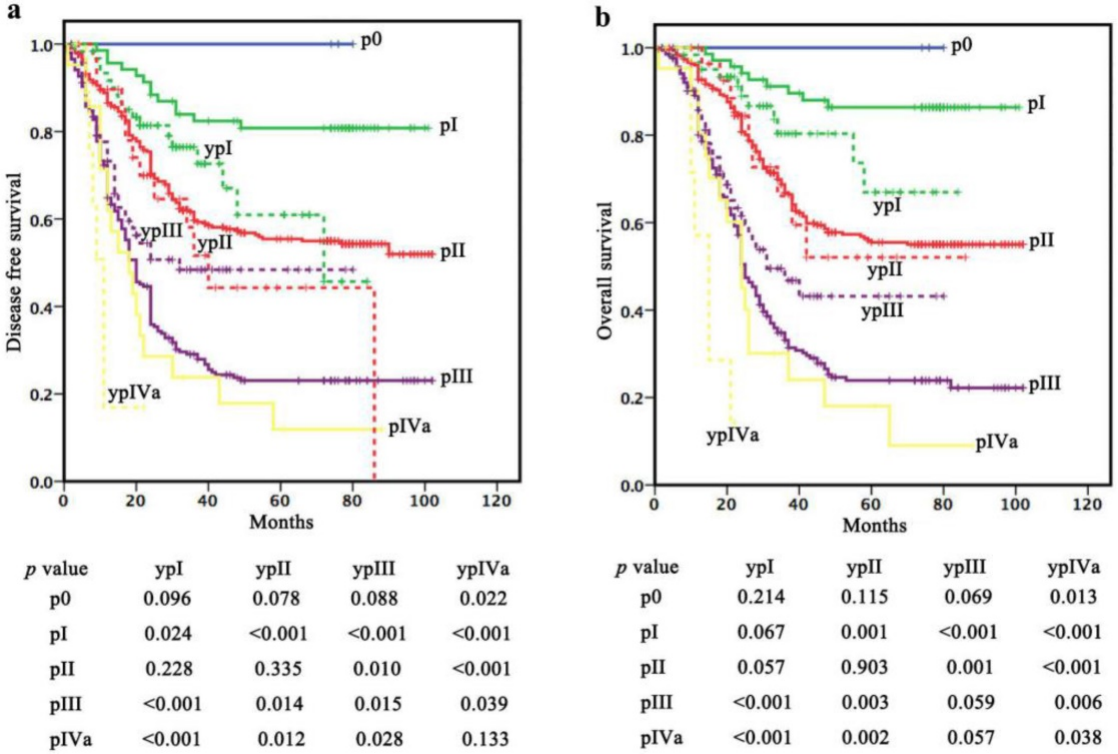
Survival comparisons within “the same stage”. Patients in ypI stage (cohort 2) had a significantly poorer DFS and a potential poorer OS compared with those in pI stage (cohort 1). There was no significant difference in DFS or OS between ypII (cohort 2) and pII (cohort 1). Patients in ypIII stage (cohort 2) had a significantly better DFS and a potential better OS compared with those in pIII stage (cohort 1). Patients in ypIVa stage (cohort 2) had a significantly poorer OS and a potential poorer DFS compared with those in pIVa stage.

Patients in ypI stage (cohort 2) had a significantly poorer DFS and a potential poorer OS compared with those in pI stage (cohort 1) (*P=*0.024 for DFS, *P*=0.067 for OS). However, no difference in survival was observed between patients in ypI (cohort 2) and pII (cohort 1) stages (*P*=0.228 for DFS, *P*=0.057 for OS). Moreover, there was no significant difference in DFS (*P*=0.335) or OS (*P*=0.903) between ypII (cohort 2) and pII (cohort 1). Patients in ypIII stage (cohort 2) had a significantly better DFS and a potential better OS compared with those in pIII stage (cohort 1) (*P*=0.015 for DFS, *P*=0.059 for OS) but a poorer survival compared with those in pII stage (cohort 1) (*P*=0.010 for DFS, *P*=0.001 for OS). Patients in ypIVa stage (cohort 2) had a significantly poorer OS and a potential poorer DFS compared with those in pIVa stage (*P*=0.038 for OS, *P*=0.133 for DFS) (Figure 2).

## Discussions

In recent years, neoadjuvant therapy has come to be included in potentially curative treatment of EC prior to surgical resection^7^, ^8^, ^20^, ^21^. The prognostic strength of new ypTNM stage based on the 8th edition of AJCC ESCC staging system needs to be evaluated^9^, ^22^. Because squamous cell carcinoma is the most common histological type of EC in China^3^, ^23^, we believed that more data from Chinese patients should be assembled to analyze the prognostic significance. However, at present, few studies have reported in China^17^. In this retrospective study, we aimed to determine the prognosis of two cohorts of patients undergoing surgery alone or receiving neoadjuvant therapy followed by surgery, and these patients staged with the recently issued 8th edition of the AJCC TNM staging system. Moreover, we elucidated the prognostic difference between ypTNM and equivalent pTNM.

### The prognosis of two cohorts

In our cohort 1 (surgery alone), the 1-, 3- and 5-year DFS rates were 79.5%, 51.1% and 46.4%, respectively, with a median survival time of 39.0 months. The 1-, 3- and 5-year OS rates were 88.9%, 57.3% and 47.6%, respectively, with a median survival time of 48.0 months. The survival rates obtained in our study were very similar with previous reports. Chen et al. have collected data from 2,011 ESCC patients who underwent surgical resection alone, and shown that the 1-, 3- and 5-year OS rates are 83.5%, 57.4% and 47.4%, respectively, with a median survival time of 51.0 months^24^.

In cohort 2 (neoadjuvant therapy plus surgery), the 1-, 3- and 5-year DFS rates were 80.4%, 57.6% and 50.3%, respectively, with a median survival time of 21.0 months. The 1-, 3- and 5-year OS rates were 91.0%, 60.9% and 51.0%, respectively, with a median survival time of 24.0 months. Consisted with our findings, Shapiro et al. have demonstrated that the 1-, 3- and 5-year DFS rates in the neoadjuvant chemoradiotherapy plus surgery group are 71%, 51% and 44%, respectively, and the 1-, 3- and 5-year OS rates are 81%, 58% and 47%, respectively^7^.

### The prognostic difference between pTNM and equivalent ypTNM

pTNM stages are the most commonly used parameters to stratify patients for prognosis after surgical resection. In 2013, Chen et al. have analyzed data with the 7th edition of AJCC staging system and reported that the 5-year survival rates in p0, pIa, pIb, pIIa, pIIb, pIIIa, pIIIb, pIIIc and pIV stages are 100%, 84.8%, 78.6%, 66.5%, 53.4%, 33.6%, 22.4%, 15.0% and 0%, respectively^24^. In 2016, Huang et al. have retrospectively analyzed the clinicopathologic data of 766 ESCC patients according to the 7th edition of AJCC staging system and suggested that the 3-year survival rates in pIa, pIb, pIIa, pIIb, pIIIa, pIIIb and pIIIc stages are 85.7%, 71.1%, 82.1%, 76.8%, 53.5%, 32.5% and 29.5%, respectively^11^. Considering the rearrangement and renaming of the 8th edition of AJCC staging system^16^, ^18^, we conducted further survival analysis in our patients with different pTNM stages using the new system. In p0, pIb, pIIa, pIIb, pIIIa, pIIIb and pIVa stages of cohort 1, our 5-year DFS and OS were 100/100%, 80.5/86.2%, 58.9/57.8%, 51.1/52.7%, 36.3/35.8%, 21.5/22.6% and 11.9/18.0%, respectively.

ypTNM staging is new to the 8th edition, and survival for ypTNM stage groups differs from that for comparable pTNM stage groups^16^, ^18^. In ypI, ypII, ypIII and ypIVa stages of our cohort 2, the 5-year DFS and OS were 60.9/67.0%, 44.3/52.1%, 48.4/43.2% and 0, respectively. Previous studies have indicated that ypTNM stage grouping is a superior predictor of outcome in stage III ESCC patients undergoing neoadjuvant therapy followed by radical esophagectomy, and patients with down-staged tumors after neoadjuvant therapy experience improved survival compared with patients without response^25^, ^26^. In our cohort 2, a total of 175 patients were evaluated with cIII-IVa stage disease before the treatment, neoadjuvant therapy was associated with significant tumor down-staging, and 92 cases (52.5%) were down-staged to ypI (62) or ypII (30).

To understand the real-world survival status of patients with different ypTNM stages, we conducted the equivalent stage comparisons between cohort 2 and cohort 1. The DFS of ypI was poorer than that of pI. There was no significant difference in survival between ypI/II and pII. The DFS of ypIII was better than that of pIII. The OS of ypIVa was poorer than that of pIVa. In WECC's data, the survival of ypTNM stage group I is equivalent to pTNM stage group IIB, but the survival for ypTNM stage group II is intermediate between pTNM stage groups IIB and IIIA, and survival for ypTNM stage groups IIIA through IVB is equivalent to the same pTNM stage groups. There were some differences between our study and WECC's conclusion partly due to the changes of the new AJCC ypTNM staging system^17^. Therefore, it is necessary to further investigate the survival difference between ypTNM and equivalent pTNM in ESCC using the 8th edition of AJCC classification in order to improve the management and counseling of EC patients receiving neoadjuvant therapy.

In the present study, we compared the survival of patients receiving neoadjuvant therapy with that of patients with equivalent pathologic categories receiving surgery alone based on separate ypTNM stage in the 8th edition of AJCC staging system. Taken together, survival of ypI was closed but not reached to equivalent pI, prognosis of ypII was reached to equivalent pII, and survival of ypIII tended to be better compared with equivalent pIII. The prognosis of ypIVa was worse compared with equivalent pIVa, indicating those patients were primary resistant to prescribed neoadjuvant therapy.

## Figures and Tables

**Table 1:** American Joint Committee on Cancer Pathologic Stage Groups and Post-neoadjuvant Pathologic Stage Groups for ESCC

Pathologic Stage Groups (pTNM)							Postneoadjuvant Pathologic Stage Groups (ypTNM)			
pT	pN	M	Grade	Location	Stage Group		ypT	ypN	M	Stage Group
Tis	N0	M0	NA	Any	0		T0-T2	N0	M0	I
T1a	N0	M0	G1	Any	IA		T3	N0	M0	II
T1a	N0	M0	G2-G3	Any	IB		T0-T2	N1	M0	IIIA
T1a	N0	M0	GX	Any	IA		T3	N1	M0	IIIB
T1b	N0	M0	G1-G3	Any	IB		T0-T3	N2	M0	IIIB
T1b	N0	M0	GX	Any	IB		T4a	N0	M0	IIIB
T2	N0	M0	G1	Any	IB		T4a	N1-N2	M0	IVA
T2	N0	M0	G2-G3	Any	IIA		T4a	NX	M0	IVA
T2	N0	M0	GX	Any	IIA		T4b	N0-N2	M0	IVA
T3	N0	M0	Any	Lower	IIA		Any T	N3	M0	IVA
T3	N0	M0	G1	Upper/middle	IIA		Any T	Any N	M1	IVB
T3	N0	M0	G2-G3	Upper/middle	IIB					
T3	N0	M0	GX	Any	IIB					
T3	N0	M0	Any	Location X	IIB					
T1	N1	M0	Any	Any	IIB					
T1	N2	M0	Any	Any	IIIA					
T2	N1	M0	Any	Any	IIIA					
T2	N2	M0	Any	Any	IIIB					
T3	N1-N2	M0	Any	Any	IIIB					
T4a	N0-N1	M0	Any	Any	IIIB					
T4a	N2	M0	Any	Any	IVA					
T4b	N0-N2	M0	Any	Any	IVA					
Any T	N3	M0	Any	Any	IVA					
Any T	Any N	M1	Any	Any	IVB					

G, histologic grade; M, metastasis classification; NA, not applicable; pN, pathologic lymph node classification; pT, pathologic tumor classification; Tis, tumor in situ. ypN, postneoadjuvant pathologic lymph node classification; ypT, postneoadjuvant pathologic tumor classification.

**Table 2:** Baseline Characteristics of the Patients

Cohort 1				Cohort 2		
	No.	%			No.	%
**Sex**				**Sex**		
Female	116	19.2		Female	27	15.4
Male	488	80.8		Male	148	84.6
**Age**				**Age**		
<60	264	43.7		<60	80	45.7
≥60	340	56.3		≥60	95	54.3
**Site**				**Site**		
Upper	27	4.5		Upper	29	16.6
Middle	327	54.1		Middle	82	46.9
Lower	250	41.4		Lower	64	36.6
**pT**				**ypT**		
Tis	4	0.7		T0	26	14.7
T1a	20	3.3		Tis	0	0
T1b	54	8.9		T1	30	17.1
T2	140	23.2		T2	36	20.6
T3	385	63.7		T3	80	45.7
T4a	1	0.2		T4a	3	1.7
**pN**				**ypN**		
N0	369	61.1		N0	92	52.6
N1	136	22.5		N1	51	29.1
N2	78	12.9		N2	23	13.1
N3	21	3.5		N3	9	5.1
**Grade**						
G1	22	3.6				
G2	338	56				
G3	240	39.7				
**pTNM**				**ypTNM**		
0	4	0.7		I	62	35.4
IA	1	0.2		II	30	17.1
IB	68	11.3		IIIA	23	13.1
IIA	175	29		IIIB	50	28.6
IIB	132	21.9		IVA	10	5.7
IIIA	23	3.8				
IIIB	180	29.8				
IVa	21	3.5				
**Disease progression**				**Disease progression**		
No	290	48		No	105	60
Yes	314	52		Yes	70	40
**Die of esophageal cancer**				**Die of esophageal cancer**		
No	306	50.7		No	116	66.3
Yes	298	49.3		Yes	59	33.7

**Table 3:** Comparison of 1-year, 3-year and 5-year DFS/OS rates among different stages according to the 8th AJCC TNM staging systems in two cohorts

	DFS/OS		
	1-year, %	3-year, %	5-year, %
**pTNM**			
0	100/100	100/100	100/100
I	95.7/100	82.4/91.2	80.8/86.4
IA	-	-	-
IB	95.6/100	82.1/91.0	80.5/86.2
II	86.5/92.7	59.7/66.8	55.4/55.5
IIA	87.9/94.2	62.2/66.2	58.9/57.8
IIB	84.7/90.7	56.4/67.5	51.1/52.7
III	64.8/80.1	29.1/33.1	23.1/23.9
IIIA	65.2/91.3	42.4/41.7	36.3/35.8
IIIB	64.8/78.6	27.4/32.1	21.5/22.6
IVa	61.9/80.2	23.8/30.1	11.9/18.0
**ypTNM**			
I	91.7/96.7	76.4/80.3	60.9/67.0
II	89.7/100	51.7/66.1	44.3/52.1
III	73.2/85.7	48.4/46.8	48.4/43.2
IIIA	87.0/91.1	62.4/56.5	62.4/56.5
IIIB	66.8/83.0	42.2/42.4	42.2/37.1
IVa	16.9/57.1	-/-	-/-

## Abbreviations

ESCC: esophageal squamous cell cancer
EC: Esophageal cancer
NCCR: National Central Cancer Registry
WECC: Worldwide Esophageal Cancer Collaboration
AJCC: American Joint Committee on Cancer
CT: computed tomography
EUS: Endoscopic ultrasound
PET: positron emission tomography
OS: Disease-specific overall survival
DFS: disease free survival
